# “Appropriate” diagnostic testing: supporting diagnostics with evidence-based medicine and shared decision making

**DOI:** 10.1186/1756-0500-7-922

**Published:** 2014-12-16

**Authors:** Julian JZ Polaris, Jeffrey N Katz

**Affiliations:** Yale Law School, 127 Wall St, New Haven, CT USA; Department of Orthopedic Surgery; Division of Rheumatology, Immunology and Allergy, Brigham and Women’s Hospital, Boston, MA USA; Department of Epidemiology, Harvard School of Public Health, Boston, MA USA

**Keywords:** Shared decision making, Patient-centered decision making, Evidence-based medicine, Diagnostic testing

## Abstract

**Background:**

Evidence-based medicine is an important approach to avoiding care that is unlikely to benefit patients in both the treatment and the diagnostic context. The medical evidence alone may not determine the most appropriate care decision. Patient interests are best served when the advantages and risks of a diagnostic test are viewed through the lens of the patient’s values. That is, the paradigm of evidence-based medicine should be complemented by the paradigm of shared decision making.

**Analysis:**

Diagnostic testing may offer physiological and psychological benefits. Clinicians should also discuss the potential harms, however, which may be physiological (e.g. radiation or scarring), psychological (e.g. anxiety), and financial (e.g. cost-sharing burdens). All three of these concerns are compounded by the risk of false positives or incidental findings that are not serious, but which require decisions about further testing or treatment.

**Conclusion:**

We suggest that patient-centered decision making around diagnostic testing involves a two-step inquiry:

1. Is the test *medically* appropriate? Does the available evidence documenting short- and long-term risk and benefits support the test for its intended use, given the patient’s characteristics and symptoms?
2. Is the test appropriate *for this patient*? Has the provider initiated a conversation about tradeoffs that helps the patient evaluate whether the balance of risks and benefits is consonant with the patient’s own values and preferences? Potential benefits and harms to consider include the physiological, the psychological, and the financial.

The Excellence in Diagnostic Imaging Utilization Act of 2013 (H.R.3705) was introduced in the United States Congress in December of 2013. If passed, the Act would do three things: (1) direct professional medical societies to define standards of appropriate use for advanced diagnostic imaging technologies; (2) require practicing physicians to consult clinical decision support tools based on those standards (though not necessarily to follow the recommendations); and (3) establish data collection mechanisms on adherence to standards and usage of the decision support tools.

Like therapeutic interventions, diagnostic tests are sometimes ordered in cases where there is little likelihood of benefit to the patient, or where the expected benefits come with the risk of potential harms [1, 2]. The Act’s response is rooted in the tenets of evidence-based medicine, seeking to reduce medically unnecessary usage of diagnostic imaging in order to improve patient welfare and potentially also reduce costs. These same concerns apply more generally to other types of diagnostic testing, such as blood tests and biopsies.

The medical evidence supporting a given clinical decision is only the first step of the clinical inquiry, however. Patient interests are best served when the advantages and risks of the test are viewed through the lens of the patient’s values. Evidence-based medicine is by no means antithetical to those goals, and is most effective when complemented by the paradigm of shared decision making. The medical community is actively working on the challenge of integrating these two decision making paradigms in order to improve the quality of care [3]. Much of this literature describes clinical decisions in general or focuses on treatments, and we aim to extend that analysis to the diagnostic context. We suggest that patient-centered decision making around diagnostic testing involves a two-step inquiry:

1. Is the test *medically* appropriate? Does the available evidence documenting short- and long-term risk and benefits support the test for its intended use, given the patient’s characteristics and symptoms?
2. Is the test appropriate *for this patient*? Has the provider initiated a conversation about tradeoffs that helps the patient evaluate whether the balance of risks and benefits is consonant with the patient’s own values and preferences?

## Two paradigms: evidence-based medicine and shared decision making

In recent decades, clinicians have faced growing pressure to base their treatment decisions on rigorous research rather than local custom. Proponents of evidence-based medicine see it as a way to address unexplained geographical variance in care, improve patient wellbeing, and potentially even curb rising health care costs [1]. The Choosing Wisely campaign has further galvanized interest by identifying tests and interventions that are often used in spite of evidence suggesting an overall lack of patient benefit [1].

A much larger and more nuanced set of scenarios involves decisions about diagnostic or therapeutic options that offer potential benefits that may or may not justify associated risks. The US Preventive Services Task Force (USPSTF) issues recommendations about who should receive screening and how often for these and other tests, and they recently updated their guidelines to recommend against both PSA screening for prostate cancer and routine mammography for breast cancer in younger women. These changes prompted an outcry from some physicians and from disease-specific advocacy groups, who noted that these guidelines rely on quantitative metrics about the “average” patient without accounting for patient-specific preferences. A 45-year-old woman whose close friend recently passed away from breast cancer may feel very differently about mammography than a 85-year-old woman with several chronic conditions who would likely opt for palliative measures even if cancer were diagnosed.

Diagnostic decision making should start with an assessment of the testing options that are clinically indicated for the patient’s symptoms, signs, and other characteristics. It should not end there, however. It has become a mantra that clinicians should “treat the patient, not the disease”. Diagnosis should be similarly holistic: we urge clinicians to “diagnose the patient, not the symptom”.

The shared decision making approach involves eliciting patient-centered goals and values in order to reach a treatment plan that is consistent with the patient’s own priorities and preferences. Currently, many informed consent conversations focus primarily on the expected benefits of the recommended course of action. This is particularly likely with medications or screenings that impart low risk compared to major surgical interventions [4]. Under a model of shared decision making, clinicians should carefully review the full range of potential benefits *and* potential harms and what they would mean for that particular patient’s wellbeing.

Information about patient-centered outcomes can help patients envision their quality of life following various interventions. In addition to the risk of medical complications, patients may want to know about expected levels of pain, or the potential for incapacitation that would undermine their ability to participate in work or daily activities. Clinicians should also be mindful of patients’ financial situations. Patients with no insurance or large cost-sharing burdens may benefit from considering upfront the out-of-pocket costs for different care options [5].

Armed with this information, some patients may wish to forego a procedure with a large price tag or impact on quality of life, choosing instead to pursue a more conservative treatment or to simply live with the underlying condition [6, 7].

## Shared decision making for diagnostic testing

These treatment-related concerns remain relevant in the diagnostic context, since testing is often a predicate to treatment decisions. Before prescribing a test, clinicians should consider talking through the possible outcomes with the patient; it may well be that the differential diagnosis is populated with conditions whose treatments are unproven, unpalatable to the patient, or identical to the recommended course of action without a definitive diagnosis (conservative treatment as part of a “wait and see” approach) [6].

Clinicians and patients should also consider together several factors specific to the diagnostic decision itself (see below list for a summary). Except in rare occasions, tests do not themselves make patients feel better. The potential benefits of testing derive from the possibility of a diagnosis being made, since an accurate assessment of patients’ complaints often facilitates effective treatment. Aside from the potential physiological benefits gained by therapeutic intervention, the patient may also experience psychological benefits: worrying about symptoms can produce stress and anxiety, [8] which can be alleviated by ruling out a serious condition or by reaching an affirmative diagnosis and establishing a care plan.

### Summary of Potential Benefits and Costs to be Considered in Shared Decision Making for Diagnostic Testing

Potential benefits of testing:

- Physiological: A symptomatic patient may receive a diagnosis, thereby enabling treatment to address the underlying condition
  - ○ Consider whether the patient would actually want to undergo the recommended treatment for the conditions in the differential diagnosis.

- Psychological: The patient may finally get an answer about worrying symptoms, or may rule out a worrisome possibility.

Potential harms of testing:

- Physiological: Some tests impart medical risks, such as radiation exposure from imaging, or complications and scarring from biopsies or other invasive testing.
- Financial: The patient may have cost-sharing burdens.
- All three of these concerns are compounded by the risk of false positives or incidental findings that are not serious, but which require decisions about further testing or treatment.

The potential harms of testing typically receive less attention in informed consent conversations, [4] but they may matter a great deal to certain patients. Many diagnostic procedures can have physiological effects, such as the radiation exposure from imaging [9] or the risk of hemorrhagic complications and scarring from a biopsy. Testing may also produce anxiety through rounds of inconclusive results, or in response to findings like tumors, which suggest the possibility of a serious condition [8]. Finally, tests may impose significant out-of-pocket financial costs on patients, [5, 6] in addition to the indirect costs of the time required to return for imaging, blood draws, or tissue samples. We note that these risks may apply even for routine diagnostic tests for which clinicians may not seek formal informed consent, such as blood draws.

These three distinct types of potential burdens are all magnified by the potential for false positives or incidental findings, [2] which require patients to make further decisions about subsequent rounds of testing and treatment.

Patients may be acutely sensitive to any or all of these potential harms. Imagine George, a single father with low income who presents with persistent sciatica. His doctor suggests lumbar MRI to check for disc protrusion. However, George faces a hefty copay, and worries about missing time from work during his imaging appointment. Even with a confirmed diagnosis of disc protrusion, George would likely opt for conservative treatment with exercises and anti-inflammatory medications over discectomy or an epidural steroid injection. These preferences reflect his limited insurance coverage, his desire to remain fit for work and parenting, and a general aversion to invasive interventions. If George’s clinician helps him fully understand the costs and benefits, he may choose to forego the MRI and simply begin conservative treatment.

We note that some health care facilities assist in arranging social services to address many of these types of patient concerns. Providers who practice shared decision making can proactively identify such needs by inquiring upfront about patient preferences. This approach is preferable to discovering addressable problems only when a patient resists the clinician’s recommendation or simply fails to complete prescribed testing and treatments.

Shared decision making has received growing attention in recent years, particularly in the emotionally fraught area of cancer screening. Even there, however, patients often report that they did not discuss the trade-offs of the full range of risks and benefits [4]. We should not rely on patients to raise these concerns unprompted, given generally low levels of health literacy and the longstanding cultural power imbalances between clinicians and patients [6]. Many patients are not informed or assertive enough to initiate conversations about harms and benefits, and will accept their doctor’s recommendation without pushback or further inquiry. We note that some patients may strongly wish to defer to their physician without a collaborative decision process. These preferences should be respected, but we advocate that all patients be offered the opportunity to engage in shared decision making, [10] except in situations where shared decisions are not possible, such as emergencies. Decision aids have been developed to educate patients and facilitate conversations with providers [4–6]. These aids could potentially be incorporated into clinical decision support tools, which have traditionally relied more on evidence-based guidance (e.g. aimed at minimizing radiation exposure) [9] than on patient-centered goals.

## Conclusion

The Excellence in Diagnostic Imaging Utilization Act relies on the tenets of evidence-based medicine to address the stubborn problem of medically inappropriate diagnostic testing decisions. Shared decision making is much harder to mandate, however, since it does not depend on externally verifiable metrics like matching a diagnosis or biomarker to a recommended treatment. Clinical decision tools can include prompts for conversations about patient-centered outcomes and financial burden, but it is ultimately up to individual providers to elicit each patient’s values and preferences to help guide decisions.

To ensure appropriate diagnostic care decisions, clinicians should begin by asking what tests are appropriate *in general* for a patient with this set of characteristics and symptoms. Clinicians should then consider which test or other course of action is appropriate for *this particular patient* in light of her personal goals and preferences. Indeed, the process of deciding on a diagnostic test requires the same consideration and patience necessary for determining a medical diagnosis itself.
